# Mouse CD8^+^NKT-like cells exert dual cytotoxicity against mouse tumor cells and myeloid-derived suppressor cells

**DOI:** 10.1007/s00262-019-02363-3

**Published:** 2019-07-05

**Authors:** Zhengyuan Li, Yiqing Wu, Chao Wang, Minghui Zhang

**Affiliations:** 1 School of Medicine, Tsinghua University, Beijing, 100084 China; 2 School of Medicine, Tsinghua University, Room B343, Haidian District, Beijing, 100084 China

**Keywords:** CD8^+^NKT-like cells, Antitumor effects, MDSC, Cytotoxicity, Granzyme B

## Abstract

Our previous work has demonstrated the high efficiency of CD8^+^ natural killer T (NKT)-like cells in killing antigen-bearing dendritic cells. To evaluate their role in the tumor microenvironment, we performed in vitro and in vivo antitumor experiments to investigate whether CD8^+^NKT-like cells could kill Yac-1 and B16 cells like NK cells and kill EL4-OVA8 cells in an antigen-specific manner like cytotoxic T lymphocytes (CTLs). Unlike NK1.1^−^CTLs, CD8^+^NKT-like cells also exhibit the capability to kill myeloid-derived suppressor cells (MDSCs) in an antigen-specific manner, indicative of their potential role in clearing tumor antigen-bearing MDSCs to improve the antitumor microenvironment. In vitro blocking experiments showed that granzyme B inhibitor efficiently suppressed the cytotoxicity of CD8^+^NKT-like cells against tumor cells and MDSCs, while Fas ligand (FasL) or tumor necrosis factor-related apoptosis-inducing ligand (TRAIL) inhibition failed to produce similar effects. Transcriptomic and phenotypic analyses of CD8^+^NKT-like cells, NK cells, and NK1.1^−^CTLs indicated that CD8^+^NKT-like cells expressed both T-cell activation markers and NK cell markers, thus bearing features of both the activated T cells and NK cells. Taken together, CD8^+^NKT-like cells could exert NK- and CTL-like antitumor effects through the elimination of both tumor cells and MDSCs in a granzyme B-dependent manner.

**Electronic supplementary material:**

The online version of this article (10.1007/s00262-019-02363-3) contains supplementary material, which is available to authorized users.

## Introduction

Natural killer T (NKT) cells, as a population of lymphocytes bearing both T and NK cell lineage markers [1], have been the focus of immunological studies for decades [2, 3]. Based on their CD1d dependency and α-GalCer reactivity, NKT cells can be divided into type I (invariant NKT [iNKT] cells), type II, and NKT-like cells [1, 4]. Development of Jα18^−/−^ mice [5] and CD1d tetramer [6] has encouraged extensive investigation of the antitumor effects by iNKT cells in both basic research and clinical trials [2, 7]. Immunologists have demonstrated that the activation of iNKT cells efficiently inhibits melanoma, thymoma, and sarcoma both in vitro and in vivo [8–10] in an interferon (IFN)-γ-dependent manner [11, 12]. An iNKT cell agonist, α-GalCer, has been recently employed in clinical practices to enhance the antitumor effects in patients with cancer [13–15]. Unlike iNKT cells, type II NKT cells are CD1d restricted but express a relatively diverse T-cell receptor (TCR) repertoire [2].

In contrast, NKT-like cells are CD1d independent and express diverse TCR repertoire, indicative of their ability to recognize antigens in a manner similar to that of conventional T cells [1]. NKT-like cells from β_2_m^−/−^ mice exhibited high cytotoxic effects on tumor cells in vitro [16]. NK1.1^+^CD8 T cells from OT-I mice performed rapid and vigorous killing of tumor cells, while the NK1.1^−^ cytotoxic T lymphocyte (CTL) population failed to exhibit potent antitumor effects, indicative of the more efficient tumoricidal effects of CD8^+^NKT-like cells [17]. Although the antitumor potential of these CD8^+^NKT-like cells has been proposed, a detailed understanding of the role that CD8^+^NKT-like cells play is insufficient.

Our previous study has suggested that CD8^+^NKT-like cells could efficiently kill antigen-bearing dendritic cells (DCs) [18]. Therefore, we speculate that since CD8^+^NKT-like cells have a high cytotoxic capability, they may also kill tumor cells. Herein, we performed in vitro and in vivo assays to demonstrate that CD8^+^NKT-like cells exert cytotoxicity against tumor cells in both a NK-like and a CTL-like manner. As CD8^+^NKT-like cells could kill antigen-bearing DCs, we investigated whether these cells could eliminate myeloid-derived suppressor cells (MDSCs) in the tumor microenvironment. As expected, we found that CD8^+^NKT-like cells could also kill MDSCs in an antigen-specific manner, suggestive of an alternative antitumor mechanism of CD8^+^NKT-like cells. Further investigation of the mechanism underlying the effects of these cells on tumor cell and MDSC killing was carried out and their NK- and CTL-like cytotoxic capabilities were evaluated to indicate the distinct immunological features of CD8^+^NKT-like cells.

## Materials and methods

### Cell lines and reagents

Murine Yac-1, B16-F10, EL4 cells as well as ovalbumin (OVA)_257–264_ peptide-expressing EL4-OVA8 cells and GFP-expressing B16-GFP and EL4-OVA8-GFP cells were cultured in RPMI-1640 medium (Gibco, MA, USA) supplemented with 10% FCS (Gibco, MA USA). Recombinant mouse cytokines, including granulocyte–macrophage colony-stimulating factor (GM-CSF), interleukin (IL)-2, IL-4, IL-6, IL-7, and IL-15, were obtained from PeproTech (NJ, USA). The fluorescent dyes used for cell staining were 5-chloromethylfluorescein diacetate (CMFDA), Hoechst 33,342, and 7-aminoactinomycin D (7-AAD; Life Technologies, MA, USA).

### Mice

Wild-type (C57BL/6 mice) and transgenic mouse strains, including mT/mG mice (B6.129(Cg)-*Gt(ROSA)26Sor*^*tm4(ACTB*-*tdTomato,*-*EGFP)Luo*^/J mice), B6.GFP mice (C57BL/6-Tg(ACTB-EGFP)1Osb/J mice), OT-I mice (C57BL/6-Tg(TcraTcrb)1100Mjb/J mice) and Simian virus 40 (SV40)-specific TCR transgenic mice (B6.Cg-Tg(TcraY1,TcrbY1)416Tev/J mice), were used for experiments at 6–10 weeks of age. To generate tdTomato fluorescence transgenic OT-I mice, female mT/mG mice were mated with male OT-I mice and their specific offspring were identified.

### Flow cytometric analysis

To analyze the lymphocyte subsets and detect their surface marker expression, the lymphocytes were stained with fluorescent antibodies against TCRβ, NK1.1, CD49b, CD8, CD25, CD27, CD44, CD62L, CD69, CD107a, CD122, CD132, CD335, killer cell lectin-like receptor G1 (KLRG1), natural killer group 2D (NKG2D), Ly49D, Ly49G_2_, Ly49H, NKG2A/C/E, and various types of TCRs (BD Pharmingen, CA, USA). Expression of granzyme B and perforin was detected following intracellular cytokine staining protocols. Flow cytometry was performed using a FACSAria II (Becton–Dickinson, CA, USA), and the data were analyzed using FlowJo software.

### Isolation and activation of CD8^+^NKT-like cells, NK cells, and NK1.1^−^CTLs

Splenocytes from OT-I mice (or SV40-specific TCR transgenic mice) were cultured in RPMI-1640 medium supplemented with 10% FCS, 50 ng/mL recombinant mouse IL-2, 10 ng/mL recombinant mouse IL-7, 50 ng/mL recombinant mouse IL-15, and DC-loaded OT-1 peptides (at 1 × 10^4^ per 10^6^ splenocytes) for 7 days. These lymphocytes were collected and stained with fluorescent antibodies. The population of CD8^+^NK1.1^+^TCRβ^+^ (CD8^+^NKT-like cells), NK1.1^+^TCRβ^−^ (NK cells) and CD8^+^NK1.1^−^TCRβ^+^ (NK1.1^−^CTLs) cells was sorted using FACSAria II flow cytometry.

### RNA-seq analysis

CD8^+^NKT-like cells, NK cells, and NK1.1^−^CTLs were isolated as described above and high-throughput transcriptome sequencing was performed at Beijing Genomics Institute. Relative intensities of all genes among CD8^+^NKT-like cells, NK cells, and NK1.1^−^CTLs were plotted as heat maps to depict their relationship. Expression profiles of the markers of T-cell activation, adhesion, and cytotoxicity as well as NK cell receptors were also shown as heatmaps.

### Immunocytochemistry

B16-F10 cells were stained with DDAO-SE (red) and co-cultured with CD8^+^NKT-like cells on slides. Fixation and permeabilization were carefully carried out using intracellular cytokine staining kit (BD Pharmingen, CA, USA) on slides at 8, 16, and 24 h. These cells were stained with Hoechst 33,342 (blue, Life Technologies, MA, USA) and phycoerythrin (PE)-conjugated antibody against granzyme B (red). Images demonstrating the interaction between B16-F10 and CD8^+^NKT-like cells were obtained by BD Pathway 855 High Content Imager.

### Isolation of MDSCs

Splenocytes from mT/mG mice were isolated and stained with allophycocyanin-conjugated antibody to CD11b and PE-conjugated antibody to Gr-1. CD11b^+^Gr-1^+^ cells were sorted as MDSCs.

### Live cell imaging

Effector cells (CD8^+^NKT-like cells, NK cells, or NK1.1^−^CTLs) from tdTomato fluorescence transgenic OT-I mice were co-cultured with B16-GFP, EL4-OVA8-GFP, or MDSCs loaded with 1 μg/mL OVA_257–264_ peptides at *E*:*T* ratios of 1:3, 1:5, or 1:10, respectively, on confocal dishes. A dynamic process showing the interaction between the effector cells (red) and target cells (green) was recorded using Andor spinning disk live cell confocal microscopy with a 40 × oil immersion lens.

### In vitro cytotoxicity assay

Target tumor cells were stained at 4 °C with CMFDA (Molecular Probes, Invitrogen) at a concentration of 1 μmol/mL for 10^6^ cells. After 10 min of incubation, cells were washed thrice with PBS containing 10% FCS. The effector cells were co-cultured in 96-well plates with 1 × 10^4^ target cells in RPMI-1640 containing 10 % FCS and 50 ng/mL of recombinant IL-2 at indicated *E*/*T* ratios. Cells were harvested every 12 or 24 h and incubated with 7-AAD (Molecular Probes, Invitrogen) at room temperature for 10 min. The cells were washed once with PBS and analyzed on BD FACSAria II. The percentage of 7-AAD-positive cells indicated the killing rate.

### In vivo adoptive transfer assay

A total of 5 × 10^4^ B16 melanoma cells or 5 × 10^6^ EL4-OVA8 thymoma cells were intravenously or subcutaneously inoculated into recipient C57BL/6 mice, respectively. Effector CD8^+^NKT-like cells, NK cells or NK1.1^−^CTLs were used for peritoneal adoptive transfer. The tumor growth and survival rates were followed and recorded at the indicated time points or at the end of experiments.

### Examination of tumor antigen-loaded MDSCs

A total of 2 × 10^6^ EL4 or EL4-OVA8 cells were subcutaneously injected. On day 10 when the tumor size reached about 1 cm^3^, mice were sacrificed and tumors were resected. The tumors were digested with 1 mg/mL of collagenase IV (Sigma) at 37 °C for 1 h. Dissociated cells were collected through a 70 μm filter and stained with allophycocyanin/Cy7-conjugated anti-CD45.2, peridinin chlorophyll protein complex (PerCP)-conjugated anti-CD11b, PE-conjugated anti-Gr-1, and allophycocyanin-conjugated anti-H_2_K^b^ bound to SIINFEKL (which could recognize the OVA_257–264_–H_2_K^b^ complex). In this assay, allophycocyanin-conjugated mouse IgG1, a κ-isotype control antibody (BioLegend), was used as an isotype control. Intratumoral MDSCs were identified as CD11b^+^Gr-1^+^ cells and their expression level of OVA_257–264_–H_2_K^b^ complex was evaluated.

### Statistical analysis

A two-tailed Student’s *t* test was used to compare two groups of normally distributed data and a Mann–Whitney *U* test was used when data were non-normally distributed. Error bars show standard errors. Difference between groups was considered statistically significant at *P* < 0.05 or less. ^***^P < 0.001, **P < 0.01, *P < 0.05, and “ns” indicated not significant.

## Results

### CD8^+^NKT-like cells kill tumor cell-like NK cells

**Fig. 1:**
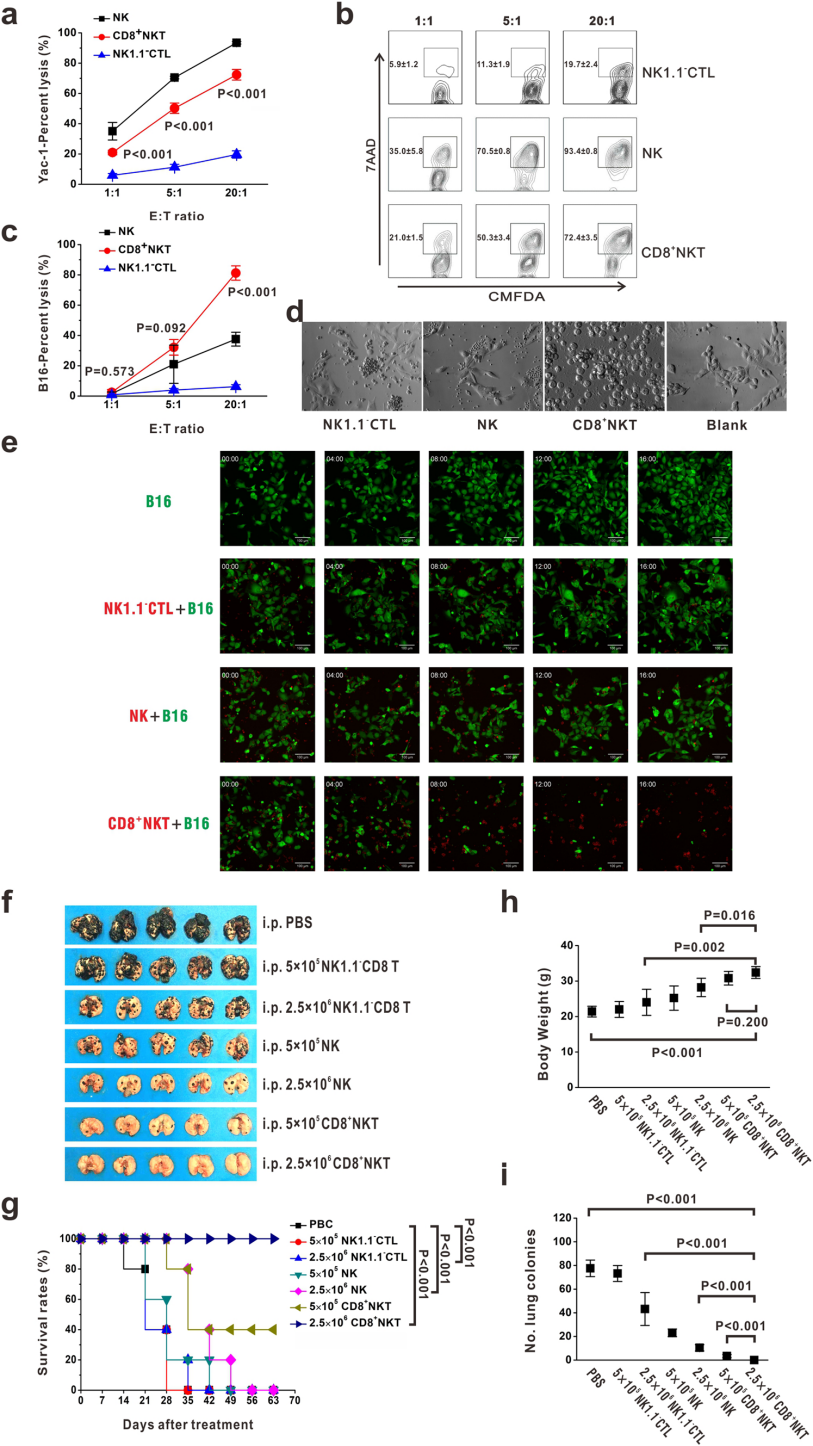
CD8^+^NKT-like cells kill tumor cells similar to NK cells. **a**, **b** CMFDA-stained Yac-1 cells were co-cultured with either CD8^+^NKT-like cells or NK cells or NK1.1^−^CTLs at *E*:*T* ratios of 1:1, 5:1, and 20:1. After 24 h, 7-AAD was added and the apoptotic cells were detected by flow cytometry. Statistical data (**a**) and the corresponding flow cytometry charts (**b)** are shown. **c**, **d** CMFDA-stained B16 cells were co-cultured with either CD8^+^NKT-like, NK cells or NK1.1^−^CTLs at *E*:*T* ratios of 1:1, 5:1, and 20:1. After 24 h, 7-AAD was added and the apoptotic cells were examined by flow cytometry (**c**). Morphology of the co-culture system at the *E*:*T* ratio of 5:1 was observed with microscopy (**d**). **e** Dynamics of the killing process of B16-GFP cells (green) by CD8^+^NKT-like cells (red), NK cells (red) or NK1.1^−^CTLs (red) are shown. **f**, **i**. A total of 5 × 10^4^ B16 melanoma cells were intravenously inoculated into C57BL/6 mice. After 12 h, 5 × 10^5^ (low-dose group) or 2.5 × 10^6^ (high-dose group) CD8^+^NKT-like cells or NK cells or NK1.1^−^CTLs were used for peritoneal adoptive transfer. Lung metastasis (**f**), survival rates (**g**), body weights (**h**), and number of lung metastatic colonies (**i**) for each group are shown. In vitro experiments were performed in triplicate and repeated three times with similar results. In vivo experiments were repeated three times. *n* = 5 for each repeat

Our previous work has demonstrated that CD8^+^NKT-like cells could exert high cytotoxicity against DCs [18]. Therefore, we investigated the ability of these cells to kill tumor cells. The NK cell-sensitive Yac-1 cells were stained with CMFDA and co-cultured with CD8^+^NKT-like cells, NK cells, or NK1.1^−^CTLs at *E*:*T* ratios of 1:1, 5:1, and 20:1. After 24 h, 7-AAD was added and the apoptotic cells were detected by flow cytometry. Our data presented that CD8^+^NKT-like cells showed a higher killing rate of Yac-1 cells than NK1.1^−^CTLs (Fig. 1a, b). B16 melanoma cells with abnormal expression of major histocompatibility complex (MHC)-I molecules (Supplementary Figure 1) were also eliminated after co-culture with CD8^+^NKT-like cells but not with NK cells or NK1.1^−^CTLs (Fig. 1c, d). The dynamics of the killing process against B16 cells by CD8^+^NKT-like cells, NK cells, or NK1.1^−^CTLs are shown in Fig. 1e. These data demonstrate that CD8^+^NKT-like cells exerted NK-like cytotoxicity against target cells upon abnormal MHC expression, while NK1.1^−^CTLs failed to inhibit Yac-1 or B16 cell growth. To confirm this observation, we examined the tumor suppression effects of these cells in B16 lung metastatic models in vivo. A total of 5 × 10^4^ B16 melanoma cells were intravenously inoculated into C57BL/6 mice. After 12 h, 5 × 10^5^ (low-dose group) or 2.5 × 10^6^ (high-dose group), CD8^+^NKT-like cells and the same amount of NK cells or NK1.1^−^CTLs were used for peritoneal adoptive transfer. We found that the adoptive transfer of NK cells or CD8^+^NKT-like cells inhibited B16 metastasis, wherein the same number of CD8^+^NKT-like cells could provide a stronger protection than NK cells (Fig. 1f, i). However, NK1.1^−^CTLs did not provide significant protection against B16 cells (Fig. 1f, i). Survival rates (Fig. 1g), body weight (Fig. 1h), and lung metastasis (Fig. 1i) were also examined and the data showed that the ability of CD8^+^NKT-like cells to inhibit B16 metastasis was the highest among the three effector cells. These results indicate that CD8^+^NKT-like cells could exert NK-like cytotoxicity against target cells both in vitro and in vivo.

### CD8^+^NKT-like cells kill tumor cells in an antigen-specific manner

**Fig. 2:**
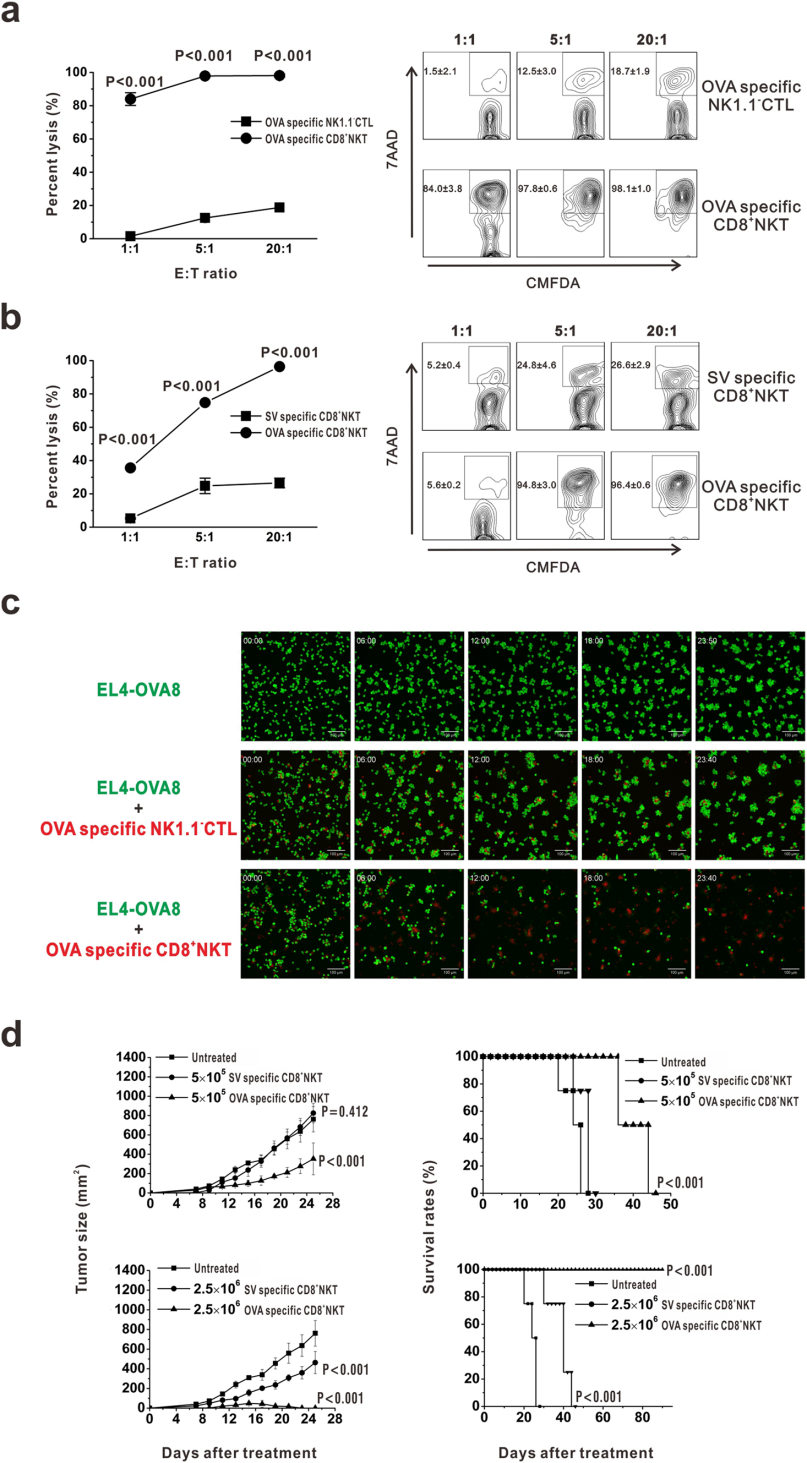
CD8^+^NKT-like cells kill tumor cells in an antigen-specific manner. **a** CD8^+^NKT-like cells and NK1.1^−^CTLs were sorted from OT-I mice and co-cultured with EL4-OVA8 cells at indicated *E*:*T* ratios. After 24 h, cells were collected and 7-AAD was added. Statistical results (left) as well as representative flow cytometry charts (right) are shown. **b** OVA or SV antigen-specific CD8^+^NKT-like cells were incubated with EL4-OVA8 cells at the indicated *E*:*T* ratios, and the killing rate of target cells was examined with 7-AAD staining at 24 h. Statistical results (left) and representative flow cytometry charts (right) are shown. **c** Dynamics of cytotoxicity of CD8^+^NKT-like cells or NK1.1^−^CTLs against EL4-GFP-OVA8 cells are shown. **d** A total of 5 × 10^5^ or 2.5 × 10^6^ OVA- and SV-specific CD8^+^NKT-like cells were adoptively transferred into 5 × 10^6^ EL4-OVA8-bearing mice. Tumor growth (left) and survival rates (right) of recipient mice were observed and recorded. In vitro experiments were performed in triplicate and repeated three times with similar results. In vivo experiments were repeated three times. *n* = 8 for each repeat

As CD8^+^NKT-like cells kill DCs in an antigen-specific manner [18], we speculated that these cells may also kill target cells in a manner observed for CTLs. Thus, we examined the effects of CD8^+^NKT-like cells on EL4-OVA8 cells in an in vitro co-culture system, wherein CD8^+^NKT-like and NK1.1^−^CD8 T (NK1.1^−^CTLs) cells were sorted from OT-I mice. After 24 h, the cells were collected and treated with 7-AAD. As a result, CD8^+^NKT-like cells showed a higher killing rate of target cells than NK1.1^−^CTLs at the indicated *E*:*T* ratios (Fig. 2a). To evaluate the antigen dependency of CD8^+^NKT-like cells to mediate tumoricidal activity, OVA or SV antigen-specific CD8^+^NKT-like cells were incubated with EL4-OVA8 cells and their killing rates were examined. As a result, OVA-specific CD8^+^NKT-like cells exerted higher cytotoxicity against EL4-OVA8 cells than SV-specific CD8^+^NKT-like cells (Fig. 2b). Figure 2c shows the dynamics of CD8^+^NKT-like cell-mediated killing of EL4-GFP-OVA8 cells. We performed an in vivo experiment to prove the antigen-specific inhibition of EL4-OVA8 cells by OVA-specific CD8^+^NKT-like cells (Fig. 2d). We also observed that the inhibitory effect on tumor growth was higher in the high-dose group treated with 2.5 × 10^6^ CD8^+^NKT-like cells than in the low-dose group (Fig. 2d). Together, we demonstrated the cytotoxic effects of CD8^+^NKT-like cells in an antigen-specific manner, as observed for CTLs.

### CD8^+^NKT-like cells kill tumor antigen-bearing MDSCs

**Fig. 3:**
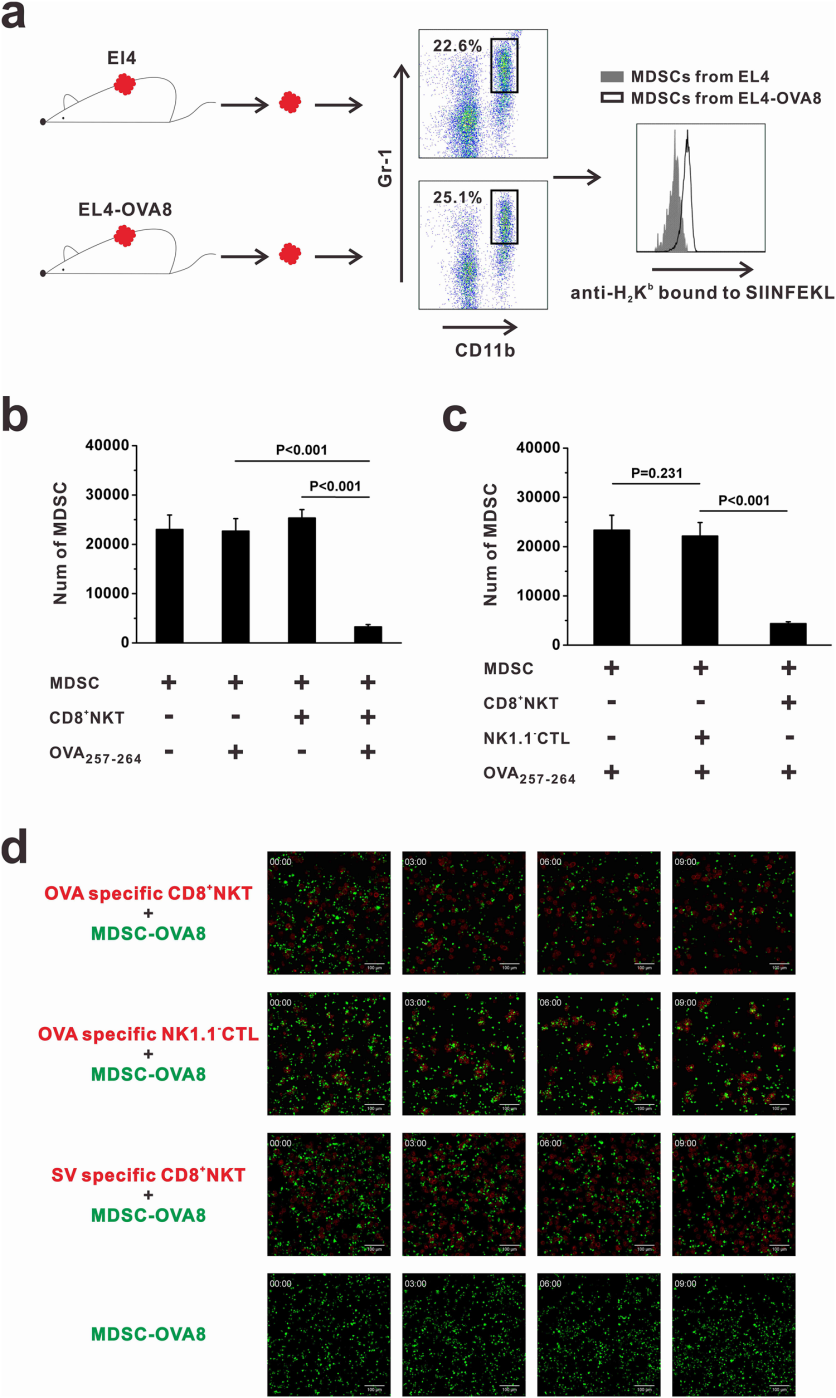
CD8^+^NKT-like cells kill the tumor antigen-bearing MDSCs. **a** Mice were subcutaneously inoculated with EL4 cells or EL4-OVA8 cells. When the tumor size reached 1 cm^3^, tumors were resected and digested with 1 mg/mL collagenase IV. Intratumoral MDSCs were identified as CD11b^+^Gr-1^+^ cells and stained with antibodies against H_2_K^b^-bound OVA_257–264_ peptides. **b** MDSCs and OVA-specific CD8^+^NKT-like cells were co-cultured at an *E*:*T* ratio of 10:1 with or without OVA peptide. After 24 h, 7-AAD-negative live MDSCs were collected and counted by flow cytometry. Relative number of live MDSCs is shown as histograms. **c** CD8^+^NKT-like cells or NK1.1^−^CTLs from OT-I mice were co-cultured with MDSCs at an *E*:*T* ratio of 10:1 with OVA peptides. After 24 h, 7-AAD-negative live MDSCs were collected and counted by flow cytometry. Relative number of live MDSCs is shown as a histogram. **d** Dynamics of the killing process of OVA_257–264_ antigen-loaded MDSCs by CD8^+^NKT-like cells or NK1.1^−^CTLs are shown. Flow cytometry analysis of OVA peptide-loaded MDSCs was performed in three independent experiments where *n* = 3 in each experiment. In vitro experiments were performed in triplicate and repeated three times with similar results

The NK- and CTL-like cytotoxicity of CD8^+^NKT-like cells against target cells suggests the ability of these cells to kill MDSCs in the tumor microenvironment. We examined whether tumor antigens could be loaded by MDSCs. The B16-OVA8 tumor model was used and flow cytometry analysis was performed. As a result, we found that intratumoral MDSCs expressed OVA_257–264_ peptides in pMHC form (Fig. 3a). We co-cultured MDSCs and OVA-specific CD8^+^NKT-like cells at an *E*:*T* ratio of 10:1 with or without OVA peptide. After 24 h, MDSCs were collected and their viability was examined by flow cytometry. In the presence of OVA peptide, MDSCs were efficiently killed by OVA-specific CD8^+^NKT-like cells. However, CD8^+^NKT-like cells could not kill MDSCs without matched antigens (Fig. 3b). We compared the killing rates of CD8^+^NKT-like cells with those of NK1.1^−^CTLs for MDSCs and found that in the presence of OVA peptides, OVA-specific CD8^+^NKT-like cells, but not OVA-specific NK1.1^−^CTLs, exerted potent cytotoxicity against MDSCs (Fig. 3c). Figure 3d shows the dynamics of the killing process of OVA antigen-loaded MDSCs mediated by CD8^+^NKT-like and NK1.1^−^CTLs. These data indicate that CD8^+^NKT-like cells killed MDSCs more efficiently than NK1.1^−^CTLs and that their function is dependent on the specific recognition of antigens.

### CD8^+^NKT-like cells exert cytotoxicity via a granule exocytosis pathway

**Fig. 4:**
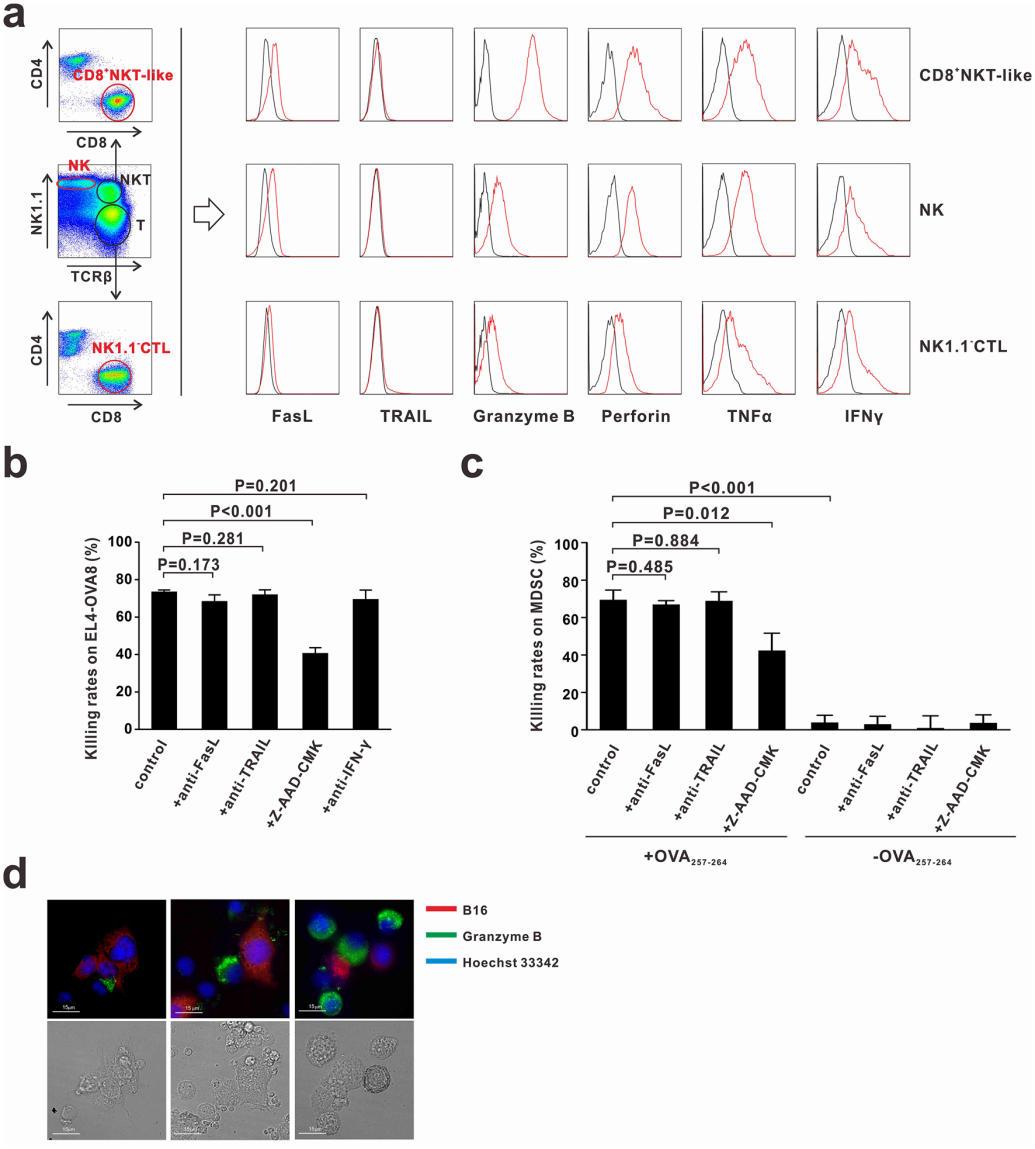
CD8^+^NKT-like cells exert their cytotoxicity via granule exocytosis pathway. **a** CD8^+^NKT-like cells, NK cells, and NK1.1^−^CTLs were gated from in vitro-activated lymphocytes and their expression of cytotoxicity-associated molecules was detected by flow cytometry. **b** OT-I mouse-derived CD8^+^NKT-like cells and EL4-OVA8 cells were co-cultured at an *E*:*T* ratio of 5:1 with or without 50 μg/mL of anti-FasL, 50 μg/mL anti-TRAIL, 50 μM granzyme B inhibitor Z-AAD-CMK, or 100 μg/mL of anti-IFN-γ. After 24 h, cytotoxicity against target cells was evaluated. **c** OT-I mouse-derived CD8^+^NKT-like cells and OVA_257–264_-loaded/unloaded MDSCs were co-cultured at an *E*:*T* ratio of 10:1 with or without 50 μg/mL anti-FasL, 50 μg/mL anti-TRAIL, or 50 μM granzyme B inhibitor Z-AAD-CMK. After 24 h, 7-AAD-negative live MDSCs were collected and counted by flow cytometry. Their cytotoxicity against target cells was evaluated and shown as a histogram. **d** Granzyme B expression was evaluated by in situ anti-granzyme B fluorescent antibody staining (green) in the co-culture system of CD8^+^NKT-like and B16 cells (red) during different killing stages. Nucleus was stained with Hoechst 33,342 (blue). These experiments were repeated thrice

To investigate the mechanisms underlying the cytotoxic effects of CD8^+^NKT-like cells against tumor cells and MDSCs, we examined the expression of cytotoxicity-associated molecules such as granzyme B, perforin, Fas ligand (FasL), tumor necrosis factor-related apoptosis-inducing ligand (TRAIL), IFN-γ, and TNF-α in CD8^+^NKT-like cells, NK cells and NK1.1^−^CTLs. Granzyme B, IFN-γ, and TNF-α expression levels were the highest in CD8^+^NKT-like cells (Fig. 4a). FasL was expressed on CD8^+^NKT-like and NK cells but not on NK1.1^−^CTLs (Fig. 4a). To determine the molecules involved in mediating the cytotoxic effects of CD8^+^NKT-like cells, we used the corresponding inhibitors (Z-AAD-CMK to inhibit granzyme B) or neutralizing antibodies (anti-FasL, anti-TRAIL, and anti-IFN-γ) in the co-culture system of CD8^+^NKT-like and target cells (EL4-OVA8 or MDSCs). As a result, we found that only granzyme B inhibitor Z-AAD-CMK efficiently suppressed the cytotoxicity of CD8^+^NKT-like cells against EL4-OVA (Fig. 4b) and MDSCs (Fig. 4c), while treatment of cells with FasL, TRAIL, and IFN-γ antibodies had no effect. To illustrate the involvement of granzyme B in the CD8^+^NKT-like cell-mediated killing process, we stained granzyme B with a fluorescent antibody (PE-labeled anti-granzyme B) in the co-culture system of CD8^+^NKT-like and B16 cells (red, stained with DDAO-SE). The staining results showed that the expression of granzyme B displayed a polarization tendency toward B16 cells (Fig. 4d). Together, these data suggest that CD8^+^NKT-like cells exert cytotoxicity against tumor cells and MDSCs through a granzyme B-mediated granule exocytosis pathway.

### Immunological features of CD8^+^NKT-like cells

**Fig. 5:**
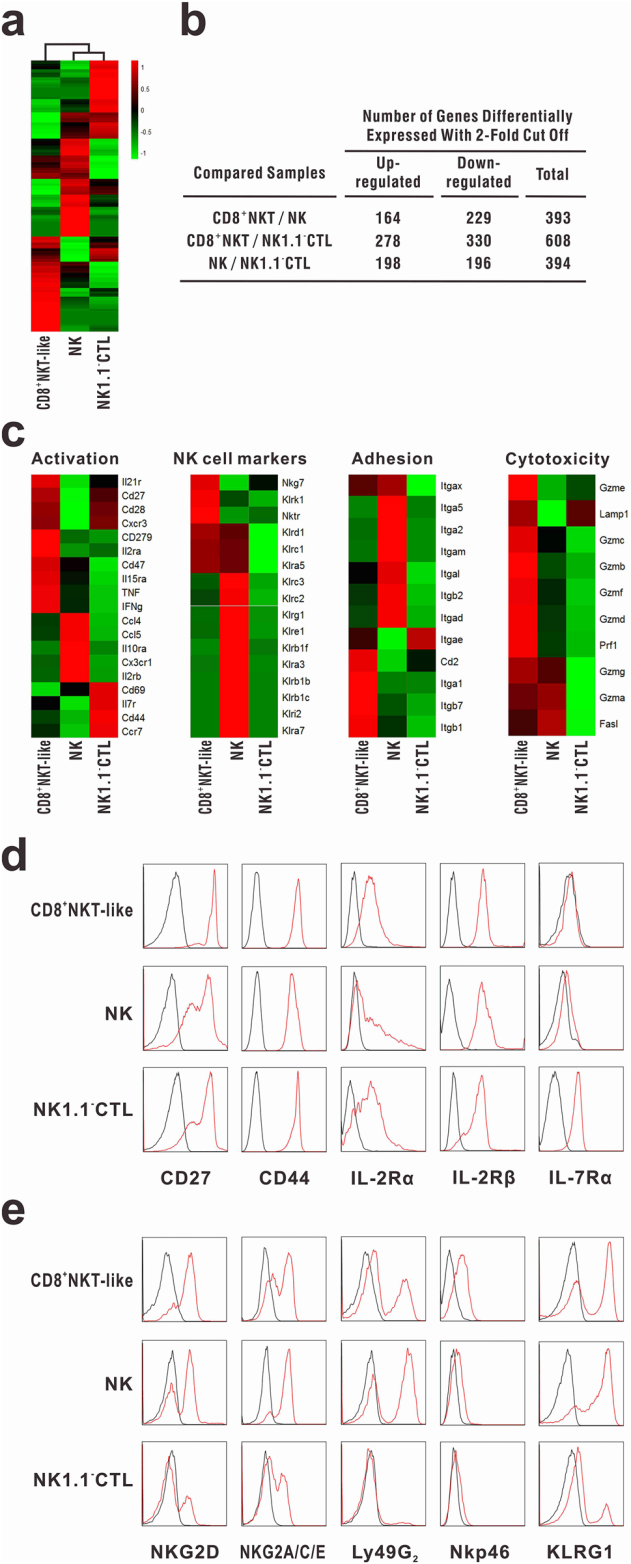
Immunological features of CD8^+^NKT-like cells. RNA-seq analysis was performed for CD8^+^NKT-like cells, NK cells, and NK1.1^−^CTLs, which were sorted from in vitro-activated OT-I mouse splenocytes (as described in “Materials and methods”). **a** Relative intensities of all genes in CD8^+^NKT-like cells with respect to NK cells and NK1.1^−^CTLs were displayed as heat maps to depict the relationship between these populations. **b** Total number of differentially expressed genes (upregulated or downregulated; twofold cutoff) between the indicated comparison groups is shown. **c** Expression profiles of the markers of T-cell activation, adhesion, and cytotoxicity as well as NK cell receptors were compared among CD8^+^NKT-like cells, NK cells, and NK1.1^−^CTLs. **d**, **e** Activated T-cell and NK cell markers were examined on CD8^+^NKT-like cells, NK cells, and NK1.1^−^CTLs by flow cytometry. Black lines show isotype controls, while red lines show the expression intensities of indicated markers. Flow cytometry experiment was repeated thrice

For the complete characterization of the population of CD8^+^NKT-like cells, genome-wide transcriptome sequencing was performed with CD8^+^NKT-like cells, NK cells, and NK1.1^−^CTLs (Fig. 5a). The genes with differential expression (upregulation or downregulation; twofold cutoff) between the indicated comparison groups are shown in Fig. 5b. The data demonstrated that the gene expression profile of CD8^+^NKT-like cells was distinct from that of NK cells and NK1.1^−^CTLs. To clarify the immunological characteristics of CD8^+^NKT-like cells, we evaluated the expression of molecules associated with lymphocyte activation, adhesion, and cytotoxicity as well as NK cell receptors (Fig. 5c) in CD8^+^NKT-like cells, NK cells, and NK1.1^−^CTLs. The results showed that CD8^+^NKT-like cells expressed both T-cell functional markers (e.g., CD8, TCR, CD44, IL-2Rα, and IL-2Rβ) and some NK cell receptors (e.g., NKG2D, NKG2A/C/E, and KLRG1), indicative of their potential function as the hybrid of CTLs and NK cells. These cells showed high expression levels of pro-inflammatory cytokines (e.g., TNF and IFN-γ) and cytokine receptors (e.g., IL-2R, IL-21R, etc.). The high expression of chemokines, chemokine receptors, and other adhesion-associated molecules in CD8^+^NKT-like cells indicated their distinct migration capability from the other two immunocytes (Fig. 5c). We found that the expression of almost all cytotoxicity-associated molecules (Fig. 5c), especially those involved in the granule exocytosis pathway, was high in CD8^+^NKT-like cells, suggesting that these cells may exert strong cytotoxicity against target cells. Flow cytometry data also demonstrated that CD8^+^NKT-like cells not only had activated T-cell phenotype (Fig. 5d) but also expressed NK cell receptors (Fig. 5e), indicating that CD8^+^NKT-like cells employ the characteristics of both NK- and CTL-like cytotoxicity while mediating antitumor effects.

## Discussion

NK cells and CTLs are the two most widely studied lymphocyte subsets in cancer immunotherapy [19–21]. Tumor antigens could be cross-presented to CTLs in an MHC-I-dependent manner [22–24], resulting in the activation of antigen-specific CTLs and efficient killing of tumor cells. However, under some circumstances, tumor cells may escape from the CTL-mediated elimination due to abnormal expression of MHC-I, mutation in tumor antigen, or MDSCs [25–27]. Unlike CTLs, NK cells could distinguish tumor cells from normal cells by recognizing the abnormal expression of MHC-I molecules or other NK cell receptor ligands [20, 21], thereby complementing the function of CTLs. Since the 1980s, adoptive cell transfer using NK cells and CTLs has been exploited in clinical trials, although with limited success [28–31]. Thus, the development of a novel technology or concept to treat cancer is needed.

The roles of CD8^+^NKT-like cells in tumor immunity are unclear because of their scarcity [4]. In our previous work, we established an in vitro method to amplify the population of CD8^+^NKT-like cells to facilitate a more comprehensive investigation of the functions of these cells [18]. In this context, we showed that CD8^+^NKT-like cells exerted potent cytotoxicity against tumor cells and MDSCs and consequently inhibited the growth of tumors. Yac-1 is an NK cell-sensitive cell line that was also found to be killed by CD8^+^NKT-like cells almost as efficiently as by NK cells; thus, CD8^+^NKT-like cells exert an antigen-independent NK-like cytotoxicity. The high expression of NKG2D on CD8^+^NKT-like cells may be responsible for this effect, as NKG2D was shown to be involved in the NK cell-mediated killing of Yac-1 cells [32, 33]. In addition, our data also show that these CD8^+^NKT-like cells kill tumor cells in an antigen-specific CTL-like manner. However, the CD8^+^NKT-like cell-mediated cytotoxicity against OVA-expressing tumor cells was higher than the NK1.1^−^CTL-mediated cytotoxicity. This phenomenon has been previously reported [17]. Thus, our data showed that CD8^+^NKT-like cells could kill tumor cells in an antigen-independent NK-like and an antigen-specific CTL-like manner.

The number of CD8^+^NKT-like cells is much smaller than that of NK cells or NK1.1^−^CTLs in naïve mice, but we demonstrated that antigen activated CD8^+^NKT-like cell responses lagged behind the response of NK1.1^−^CTLs in our previous work [18]. Therefore, although in the late contraction stage of an immune response, a considerable number of CD8^+^NKT-like cells was still maintained and started to take over the roles of CTLs in immune responses and homeostatic maintenance [18]. Based on this observation, we suggest that distinct from NK cells and NK1.1^−^CTLs that act as the first line of defense against tumors, the small population of CD8^+^NKT-like cells with more powerful antitumor capabilities may function as the second line of defense. Thus, it is also reasonable to suggest that the adoptive transfer of these CD8^+^NKT-like cells may provide efficient protection against tumors, as described in this context.

Myeloid-derived suppressor cells are considered as immunosuppressive lymphocytes in many physical and pathogenic processes and promote the escape of cancer cells from immune attacks [34, 35]. Some groups have developed approaches to eliminate or modulate the functions of these cells in vivo [36]; however, they remain resistant to cell-mediated lysis. Herein, we found that CD8^+^NKT-like cells exerted cytotoxicity against MDSCs in an antigen-specific manner, which has the potential to restore antitumor immunity. This observation was initially confusing with regard to our previously published study showing that CD8^+^NKT-like cells can kill antigen-bearing DCs to reduce inflammation. However, given the chronic inflammatory state often associated with tumorigenesis [37, 38], our findings are consistent and show that CD8^+^NKT-like cell-mediated cytotoxicity can regulate inflammation and restore homeostasis.

**Fig. 6:**
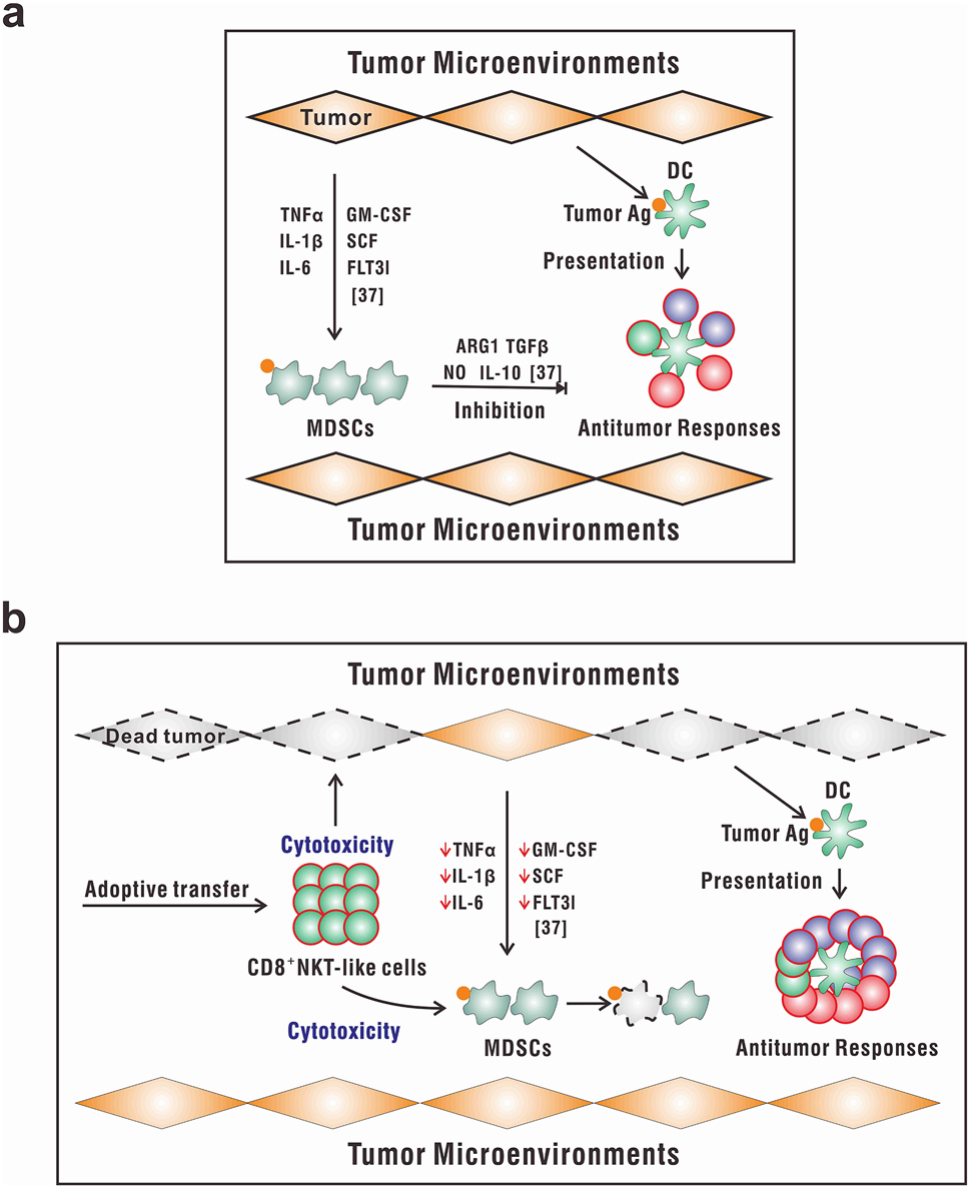
Schematic illustration of the role of CD8^+^NKT-like cells in the tumor microenvironment. **a** In the tumor microenvironment, the increased number of MDSCs produce arginase I, TGF-β, NO, or IL-10, leading to the inhibition of antitumor immune responses. **b** The adaptive transfer of CD8^+^NKT-like cells may result in the killing of tumor cells and tumor antigen-loaded MDSCs, thereby leading to an improvement of the antitumor microenvironment and elimination of tumors

Based on their cytotoxicity on tumor cells as well as MDSCs, CD8^+^NKT-like cells might play a role as direct killers and as indirect regulators of the tumor microenvironment. As shown in Fig. 6a, the increased population of MDSCs in deteriorating tumor microenvironment could produce arginase I, TGF-β, nitric oxide (NO), or IL-10 [39], eventually leading to the expansion of regulatory T cells [40] and T-cell cycle arrest [41] as well as impaired T-cell migration [42]. All these mechanisms result in the inhibition of antitumor immune responses. However, the adoptive transfer of CD8^+^NKT-like cells (Fig. 6b) resulted in the killing of tumor cells and tumor antigen-loaded MDSCs, thereby abrogating the immunosuppressive signals and contributing to the improvement of the antitumor microenvironment as well as elimination of tumors. These dual antitumor effects of CD8^+^NKT-like cells indicate their potential as an immunotherapeutic strategy. Therefore, a phase I/II clinical trial using CD8^+^NKT-like cells to treat patients with advanced melanoma is now conducted in the Beijing Cancer Hospital (NCT02619058) to further explore their antitumor effects in the clinic.

### Electronic supplementary material

Below is the link to the electronic supplementary material.

Supplementary material 1 (PDF 737 kb)

## Abbreviations

ATCC: American Type Culture Collection
B16-GFP: GFP-expressing B16 cells
CD8^+^NKT-like cells: CD8-expressing natural killer T-like cells
CMFDA: 5-chloromethylfluorescein diacetate
CTL: Cytotoxic T lymphocytes
DC: Dendritic cells
EL4-OVA8: EL4 cells transfected with OVA_257–264_ peptide
EL4-OVA8-GFP: EL4 cells transfected with both OVA_257–264_ peptide and GFP
FasL: Fas ligand
GM-CSF: Granulocyte–macrophage colony-stimulating factor
IFN: Interferon
IL: Interleukin
iNKT cells: Invariant natural killer T cells
KLRG1: Killer cell lectin-like receptor G1
MDSCs: Myeloid-derived suppressor cells
NK1.1^−^CTLs: NK1.1 negative cytotoxic T lymphocytes
NKG2D: Natural killer group 2D
NKT cells: Natural killer T cells
OVA: Ovalbumin
RNA-seq: RNA sequencing
TCR: T-cell receptor
α-Galcer: Alpha-galactosylceramide
β_2_m^−/−^: β_2_ microglobulin deficient
7-AAD: 7-Aminoactinomycin D
