# Supplementary material for: Mouse CD8+NKT-like cells exert dual cytotoxicity against mouse tumor cells and myeloid-derived suppressor cells
Source: Cancer Immunol Immunother. 2019 Jul 5;68(8):1303–15. doi: 10.1007/s00262-019-02363-3 (PMC6682577; doi:10.1007/s00262-019-02363-3)
Supplement: Supplementary file 1 — Supplementary material 1 (PDF 737 kb) [file 262_2019_2363_MOESM1_ESM.pdf]

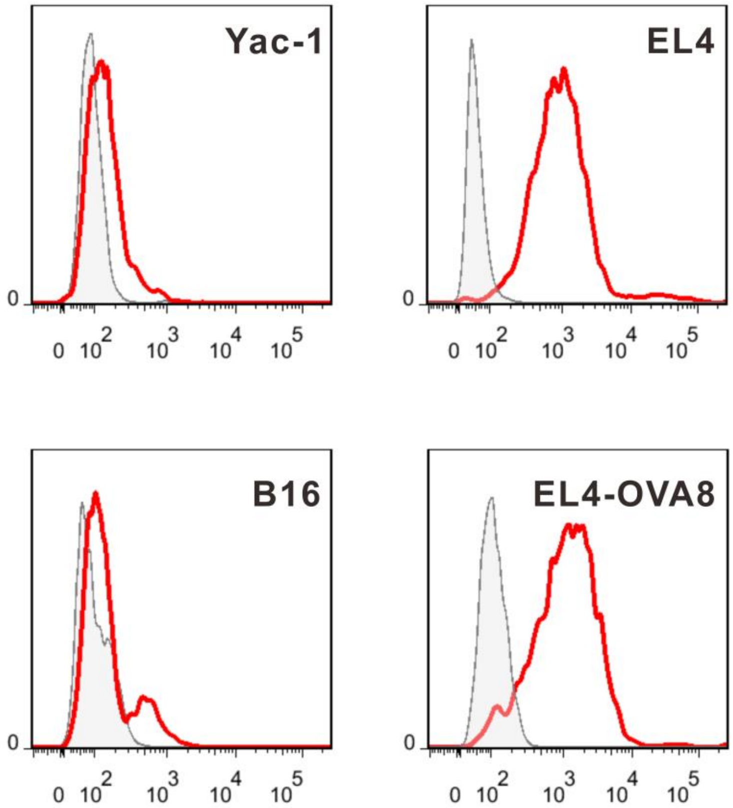

Supplementary Figure 1. MHC-I expression detection on different tumor cell lines.

Grey filled histograms show isotype controls, while red solid lines show the expression of H_2K^b on indicated tumor cell lines.

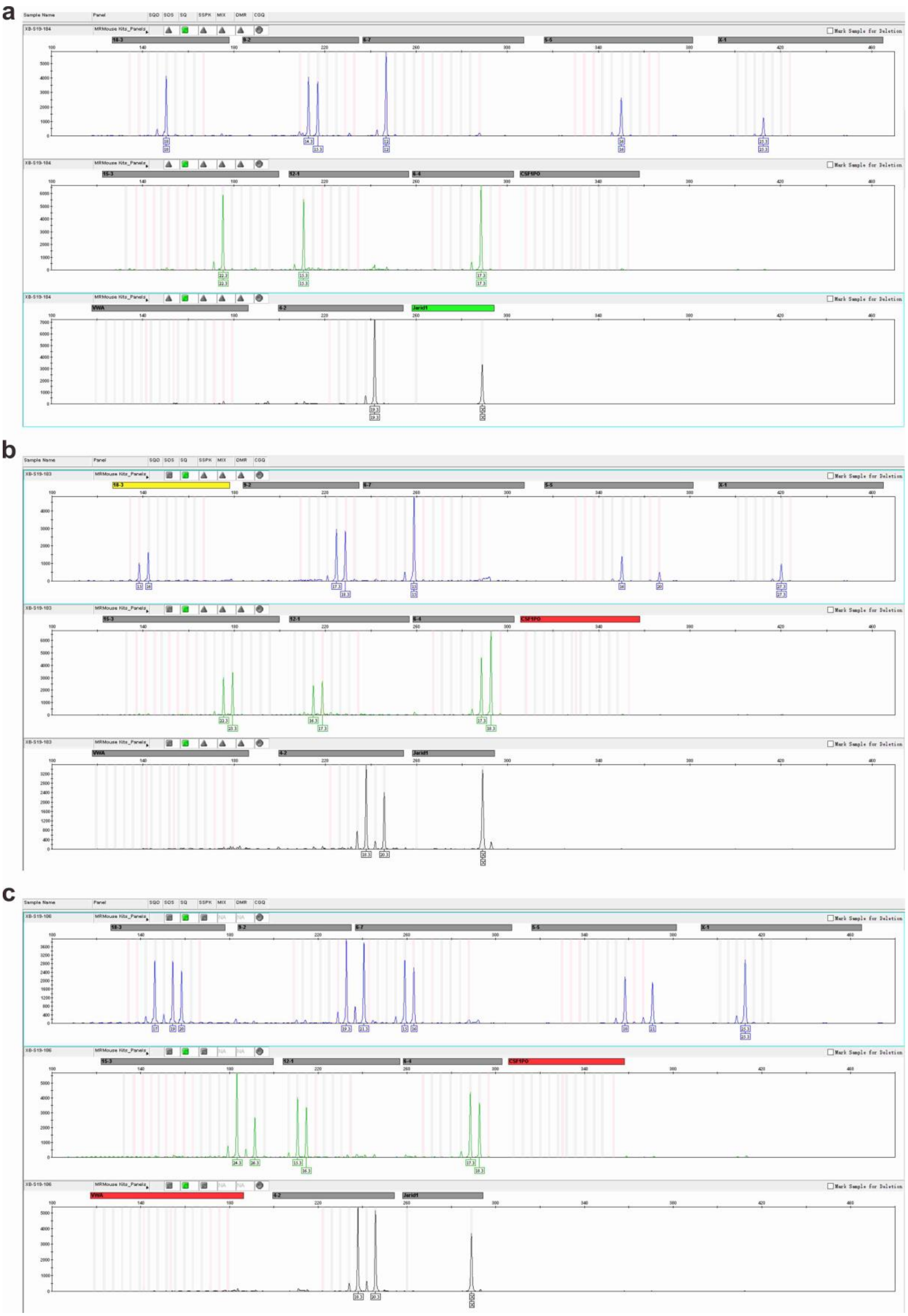
a
b
c
Sample Name
Panel
SQO SOS SQ SSPK MIX DMR CGQ
MRMouse Kits_Panels
Mark Sample for Deletion
18-3
9-2
6-7
5-5
X-1
15-3
12-1
6-4
CSF1PO
VWA
4-2
Jarid1

Supplementary Figure 2. STR profiles of murine cell lines.

PCR amplification was performed using STR Multi-amplification Kit (Mouse STR v2.0) and PCR products were assayed with 3730xl DNA Analyzer (Applied Biosystems®). STR profiles of murine Yac-1 (a), B16-F10 (b) and EL4 (c) were shown.

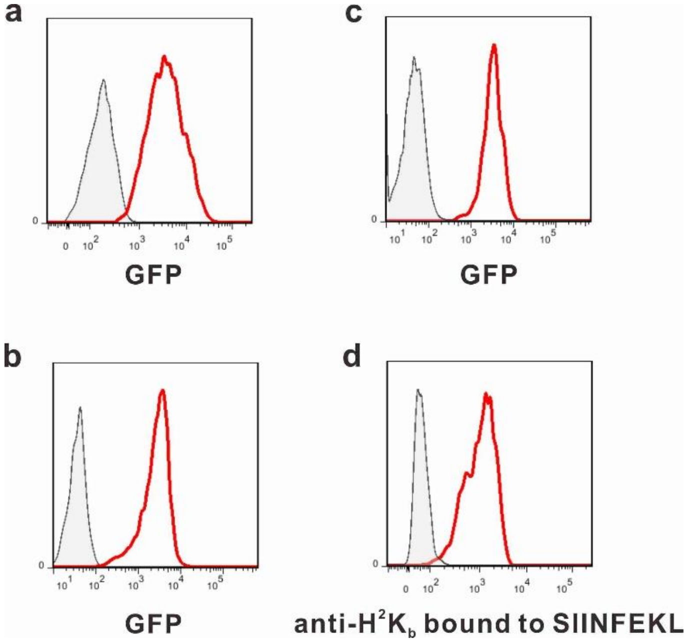

Supplementary Figure 3. GFP/OVA$_{257\text{-}264}$ Expression of transfected murine cell lines.

a. GFP expression on B16-GFP cells (red line) and B16-F10 control cells (grey histogram). *b*. GFP expression on EL4-GFP cells (red line) and EL4 control cells (grey histogram). *c*. GFP expression on EL4-OVA8-GFP cells (red line) and EL4 control cells (grey histogram). *d*. Detection of OVA$_{257\text{-}264}$ expression using anti-H^2K_b bound to SIINFEKL on EL4-OVA8-GFP cells (red line) and EL4-GFP (grey histogram) cells.
